# Performance Evaluation of Five Real‐Time PCR Assays for the Detection of *Candida auris* DNA

**DOI:** 10.1111/myc.70065

**Published:** 2025-05-03

**Authors:** Jochem B. Buil, Bart van den Bosch, Suzan J. van der Maas, Eelco F. J. Meijer, Theun de Groot, Joseph Meletiadis, Paul E. Verweij, Willem J. G. Melchers, Suzan D. Pas

**Affiliations:** ^1^ Department of Medical Microbiology Radboudumc Nijmegen the Netherlands; ^2^ Radboudumc‐CWZ Center of Expertise for Mycology Nijmegen the Netherlands; ^3^ Department of Medical Microbiology and Immunology Canisius Wilhelmina Hospital Nijmegen the Netherlands; ^4^ Clinical Microbiology Laboratory Attikon University Hospital, Medical School, National and Kapodistrian University of Athens Athens Greece; ^5^ Department of Medical Microbiology and Infectious Diseases Erasmus MC Rotterdam the Netherlands; ^6^ Unit for Mycology Statens Serum Institute Copenhagen Denmark; ^7^ Center for Infectious Disease Research, Diagnostics and Laboratory Surveillance National Institute for Public Health and the Environment (RIVM), Bilthoven the Netherlands

**Keywords:** *Candida*, *Candida* spp, PCR

## Abstract

**Objectives:**

This study aimed to systematically evaluate and compare the performance of two laboratory‐developed assays (LDAs) and three commercially available real‐time PCR assays for the detection of *Candida auris*. The analytical sensitivity, specificity and limit of detection (LOD) of each assay were assessed, alongside their clinical sensitivity in identifying *C. auris* colonisation.

**Methods:**

Ten *C. auris* strains representing five clades, as well as genetically related yeasts, common yeast species, and dermatophytes, were used to assess assay sensitivity and cross reactivity. Clinical and environmental samples were collected from patients during an outbreak and tested with three commercial PCR assays (AurisID, Fungiplex, FungiXpert) and two LDAs (CDC LDA, EMC LDA). LOD was determined using Probit analysis. Diagnostic sensitivity was evaluated by comparing the detection rate of each individual assay to the total detection rate of all assays combined.

**Results:**

The EMC LDA exhibited the highest analytical sensitivity, with a LOD of 8 conidia/reaction, followed by CDC LDA (16 conidia/reaction), AurisID and FungiXpert (19 conidia/reaction), and Fungiplex (596 conidia/reaction). Specificity testing revealed cross‐reactivity in the CDC LDA and AurisID assays with *C. pseudohaemulonii* at high conidia levels, while no cross‐reactivity was observed in the other assays. EMC LDA showed the highest clinical sensitivity (100%), whereas Fungiplex had the lowest positivity rate (71%). No false positives were observed in negative control swabs for any assay.

**Conclusions:**

Real‐time PCR is a crucial tool for the rapid and sensitive detection of *C. auris* , especially in clinical settings where timely identification is essential for effective patient management and infection control. Numerous PCR assays are available for this purpose; however, our study demonstrates that the sensitivity of these assays can vary significantly. The observed differences underscore the importance of establishing international reference standards and proficiency panels to enhance the accuracy and comparability of assay performance across different studies and laboratories.

## 1 Introduction

*Candida* (*Candidozyma*) *auris* is a multidrug‐resistant fungal pathogen that presents a significant threat to global healthcare systems due to its extensive antifungal resistance and its ability to cause outbreaks in healthcare settings [1, 2, 3]. The yeast was first described in 2009 when it was isolated from the ear canal of a patient in Japan [4]. Since then, it has spread rapidly across all continents [5]. Three genetically unrelated *C. auris* clades simultaneously emerged in different geographical regions [6]. Since then, two additional clades have been confirmed [7, 8], and a sixth clade has recently been discovered in Singapore [9].

Colonisation with *C. auris* is often asymptomatic, facilitating its nosocomial transmission and contributing to challenging outbreaks in healthcare settings. Furthermore, due to its high salt‐ and thermotolerance and ability to form a biofilm, *C. auris* effectively colonises the skin and rapidly spreads in the environment, traits unique to *C. auris* [10, 11]. Common colonisation sites include the axilla, groin and—less often—the nares. 5%–10% of colonised patients may develop invasive infections [12]. Risk factors for *C. auris* infection include immunosuppression, prolonged hospitalisation, invasive medical procedures and exposure to broad‐spectrum antibiotics or antifungal agents. Blood infections are associated with a mortality rate ranging from 30% to 72% [13]. *C. auris* is often resistant to multiple antifungal agents, including triazoles and amphotericin b and, to a lesser extent, echinocandins [14], emphasising the importance of timely and accurate detection for effective patient management and infection prevention control [3, 15].

Real‐time polymerase chain reaction (PCR)‐based assays provide rapid and sensitive detection of *C. auris* colonisation and infection, making them an invaluable tool for diagnosis, surveillance, and outbreak control when used alongside traditional mycological culture [16, 17]. Several commercially available and laboratory‐developed assays (LDAs) exist for this purpose. Although previous studies have evaluated individual PCR tests and assays for the detection of *C. auris* DNA using various methods, direct comparisons between these assays are limited. Moreover, methodological variations across studies hinder the comparability of results.

In this study, we systematically evaluated and compared the performance of two LDAs and three commercially available real‐time PCR assays for the detection of *C. auris* .

## 2 Materials and Methods

### 2.1 Sample & Strain Collection

#### 2.1.1 Strains

To study sensitivity and cross reactivity with other fungi, 10 *C. auris* strains from five clades (Table S1) were selected (for genotype inclusivity) and genetically related yeasts (*Candida haemulonii*, *Candida duobushaemulonii*, *Candida pseudohaemulonii*), seven common yeast species ( *Candida albicans* , *Candida parapsilosis* , *Pichia kudriavzevii*, *Kluyveromyces marxianus*, *Geotrichum candidum*, *Tardiomyces blankii*, *Clavispora lusitaniae*) and two dermatophytes (*Trichophyton rubrum* and *Trichophyton interdigitale*), all commonly found on skin, were selected for genotype exclusivity. All isolates were selected from the Radboudumc fungal culture collection. Yeasts were identified using MALDI‐TOF MS (Bruker), and dermatophytes by sequence analysis of the full internal transcribed spacer (ITS) region.

#### 2.1.2 Patient and Environmental Samples

Twelve eSwabs (Copan) from culture‐proven positive patients were selected, including two combined axilla/groin swabs, swabs from the nose, throat, rectum and seven wound sites. Additionally, nine axilla/groin swabs were collected from nine patients in an ICU during an ongoing *C. auris* outbreak. As proof of principle for usage in a hospital infection prevention setting, 17 environmental swabs from the rooms of patients with confirmed *C. auris* colonisation were collected. All patient and environmental samples were cultured on Sabouraud dextrose agar at 40°C for 5 days to select for *C. auris* [11]. As negative controls, 40 environmental samples from patient rooms without prior exposure to *C. auris* positive cases were collected. The three commercial and two laboratory‐developed real‐time PCR assays were tested once against these samples. Sampling of patients was approved by the Institutional Review Board of Attikon University Hospital under protocol number MIKPO EBΔ 226.

#### 2.1.3 Commercial Real‐Time PCR Assays

Three commercially available assays were used in this study, namely the qPCR Kit AurisID (OLM diagnostics) (AurisID), the Fungiplex *Candida auris* Real‐Time PCR Kit (Bruker) (Fungiplex) and the FungiXpert *Candida auris* Molecular Detection Kit (Genobio) (FungiXpert) (Table 1).

**TABLE 1:** Technical characteristics^a^ of the real‐time polymerase chain reaction assays for the detection of *C. auris* used in this study.

Assay	Nucleic acid extraction^a^	Extraction input (μL)	Extraction output (μL)	Target	PCR machines	PCR input (μL)	PCR total volume (μL)	Detection label	Sample type	Internal control	LOD	Reference
CDC LDA	Manual (freeze, heat and beadbeating)	200	200	ITS2	ABI 7500 Fast (Applied biosystems)	5	20	ITS2 (FAM) IC (LC610)	Patient swabs environmental sponges	Internal sample control (*bicoid* gene)	1 CFU/reaction	[15]
Fungiplex (Fungiplex *Candida auris* RUO Real‐Time PCR Kit) Bruker (RUO)	Qiagen EZ1 DSP Virus Kit	400	60	Not publicly available	ABI 7500 Fast/CFX96/Rotor‐Gene Q 5plex HRM/LightCycler 480 II/QuantStudio 5/Mic qPCR Cycler/FluoroCycler XT	10	25	IC (FAM) *C. auris* (CY5)	Whole blood plasma serum	Internal extraction control	9 copies/reaction	IFU and [18]
FungiXpert ( FungiXpert—*Candida auris* Molecular detection kit) Genobio (CE IVD)	Qiagen QiaAmp DNA mini kit	200	50	ITS2	“systems detecting FAM/ROX” ABI 7500 Fast/QuantStudio	5	20	ITS2 (FAM) RNAseP (ROX)	Directly from samples without culture	Internal sample control (RNAseP)	500 copies/mL	IFU
EMC LDA	MP96 (Roche)	200	50	ITS	LC480‐II (Roche)	5	20	ITS (FAM) IC (LC610)	patient swabs	internal extraction control	4 cells/reaction	[17]
AurisID (OLMdiagnostics) (CE IVD)	Any extraction protocol	200	50	Not publicly available	Systems detecting FAM/ROX	6	14	*C. auris* (FAM) IC (ROX)	Fungal cultures clinical blood samples	Internal extraction control	1 copy/μL	IFU and [18]

^a^ As stated in instructions for use (IFU) or publication.

#### 2.1.4 Laboratory‐Developed Real‐Time PCR Assays

Two laboratory‐developed real‐time PCR assays were used in the study. The first was described by Leach et al. and adopted by the CDC (CDC LDA) [15]. The second was recently described by Leonard et al. (EMC LDA) [17]. The details of the laboratory‐developed assays are shown in Table 1.

#### 2.1.5 DNA Extraction and Amplification

Fungal strains were cultured on Saboraud dextrose agar at 29°C. Conidia suspensions were added to bead‐beating tubes and DNA from isolates was extracted by incubating samples at 90°C for 10 min, followed by bead‐beating (twice for 20 s at 6500 rpm) as a pretreatment. For the eSwabs, 200 uL was similarly pretreated with bead‐beating. Subsequently, 200 uL total nucleic acids were extracted using the MagNA Pure 96 system (Roche diagnostics) using DNA/Viral NA SV 2.0 and viral NA plasma protocol with an extraction volume of 50 uL. Negative, positive, and internal controls (IC), including Phocine herpes virus/Equine arthritis virus (in‐house controls), Ext/PCR Control from the Fungiplex *Candida auris* kit, and IC from the FungiXpert *Candida auris* Molecular Detection Kit, were added to ensure the accuracy of the complete process (Table 1). Real‐time PCR amplification was performed using the LightCycler 480 Instrument II (Roche), using second derivative analysis method for in‐house assays, and following the manufacturer's instructions for each assay.

#### 2.1.6 Limit of Detection (LOD)

One *C. auris* strain, Clade I (M.072–38), was used for the LOD experiments. A conidia suspension of approximately 0.5 McFarland was prepared in sterile water by sampling a *C. auris* yeast colony grown on Sabouraud dextrose agar with a sterile swab and adjusted to a concentration of 750,000 conidia/mL by counting with a Bürker‐Türk counting chamber and adjusting the concentration accordingly. Subsequently, a dilution series was prepared resulting in an inoculum with 75,333 conidia/mL, 7533 conidia/mL, 753 conidia/mL, 377 conidia/mL, 188 conidia/mL, 94 conidia/mL, and 7.5 conidia/mL. All inoculates were tested in triplicate.

### 2.2 Diagnostic Sensitivity and Specificity

Analytical sensitivity, cross reactivity and specificity was calculated for each PCR assay. DNA from 10 *C. auris strains*, and 12 related yeast species and common dermatophytes, was tested across all PCR assays. Clinical sensitivity and specificity was calculated for each PCR assay from 23 patient samples and 17 environmental samples. Since real‐time PCR is a more sensitive detection technique than culture, there is no optimal gold standard to accurately calculate clinical sensitivity and specificity. Therefore, we defined the golden standard as ‘ *C. auris* detected’, if any of the real‐time PCRs or culture yielded a positive result.

#### 2.2.1 Statistical Analysis

LOD was determined with Probit analysis as the conidia concentration, at which the detection probability equals 95%. Probit was performed using IBM SPSS Statistics v29.

## 3 Results

### 3.1 Analytical Sensitivity and Specificity

**FIGURE 1:**
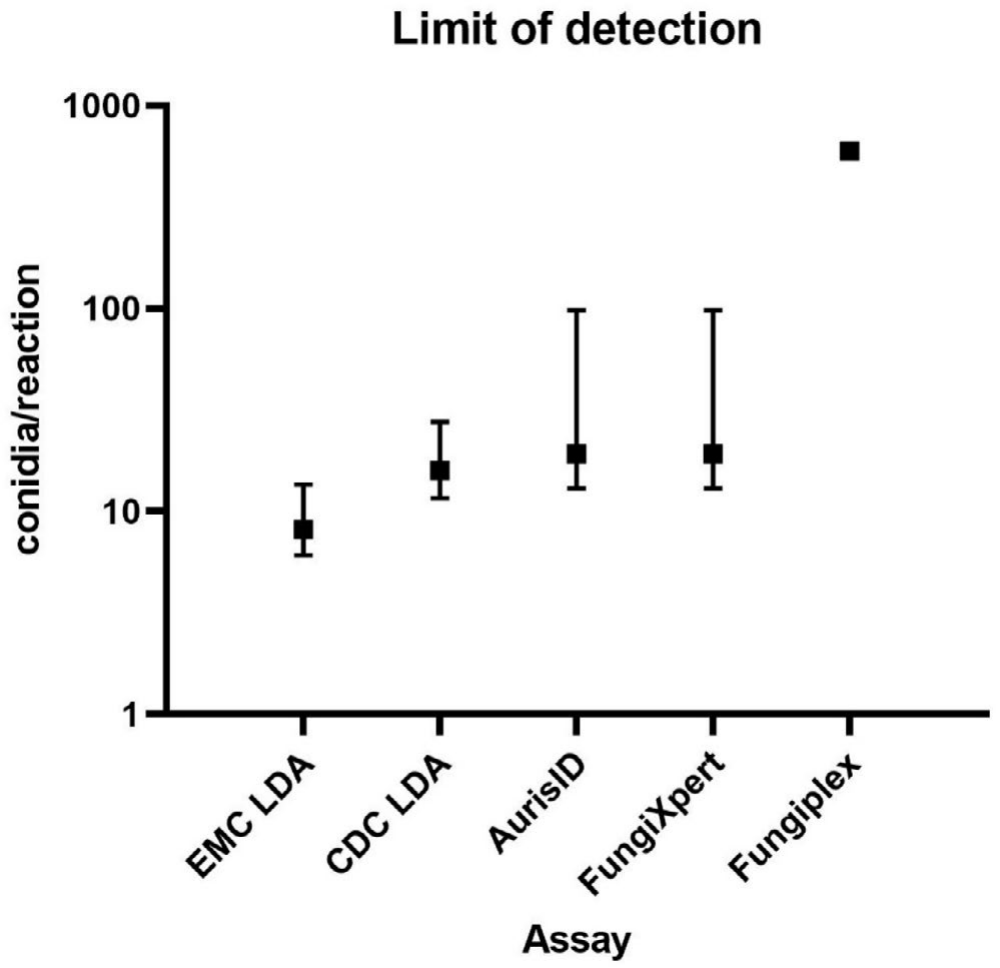
Limit of detection analysis of *C. auris* PCR. Limit of detection is displayed with a 95% confidence interval. Due to low positivity, no 95% confidence interval could be calculated for the Fungiplex assay.

*C. auris* DNA from all clades was consistently detected by all real time PCRs (Table S1). However, while no cross‐reactivity was observed for the LDA EMC, Fungiplex, and Genobio assays, both the CDC LDA and OLM AurisID assays exhibited cross‐reactivity with *C. auris* closely related *C. pseudohaemulonii*. For the CDC LDA assay, the crossing point (Cp) values for *C. auris* ranged from 14.18 to 16.36, while *C. pseudohaemulonii* had a Cp‐value of 37.76. In the OLM AurisID assay, Cp‐values for *C. auris* ranged from 13.91 to 18.55, with *C. pseudohaemulonii* showing a Cp‐value of 35. DNA from other fungal species did not result in any cross‐reactivity, demonstrating the test's overall specificity for *C. auris* . The LOD experiments indicate variability in assay sensitivity, with EMC LDA being the most sensitive and Fungiplex the least sensitive (Figure 1). Specifically, the EMC LDA *Candida auris* qPCR had a LOD of 8 conidia/reaction (95% CI: 6079–13,558 conidia/reaction). The CDC LDA had a LOD of 16 conidia/reaction (95% CI: 11,603–27,667 conidia/reaction). Both the AurisID and FungiXpert *Candida auris* PCR assays had a LOD of 19 conidia/reaction (95% CI: 12,972–98,236 conidia/reaction). The Fungiplex had a LOD of 596 conidia/reaction. In conidia/mL, the results would be 407 conidia/mL (95% CI 304–678 conidia/mL), 795 conidia/mL (95% CI: 580–1383 conidia/mL), 960 conidia/mL (95% CI: 649–4911 conidia/mL), 960 conidia/mL (95% CI: 649–4911 conidia/mL), and 29,816 conidia/mL for EMC LDA, CDC LDA, AurisID, FungiXpert and Fungipex, respectively.

### 3.2 Clinical Sensitivity

**FIGURE 2:**
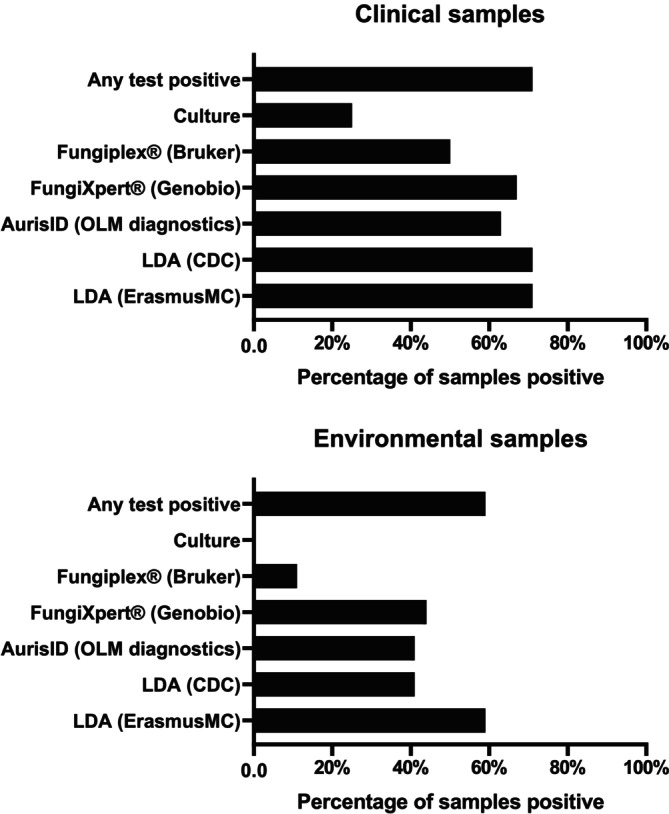
PCR positivity of patient and environmental samples and negative controls compared to any positive test and culture.

**FIGURE 3:**
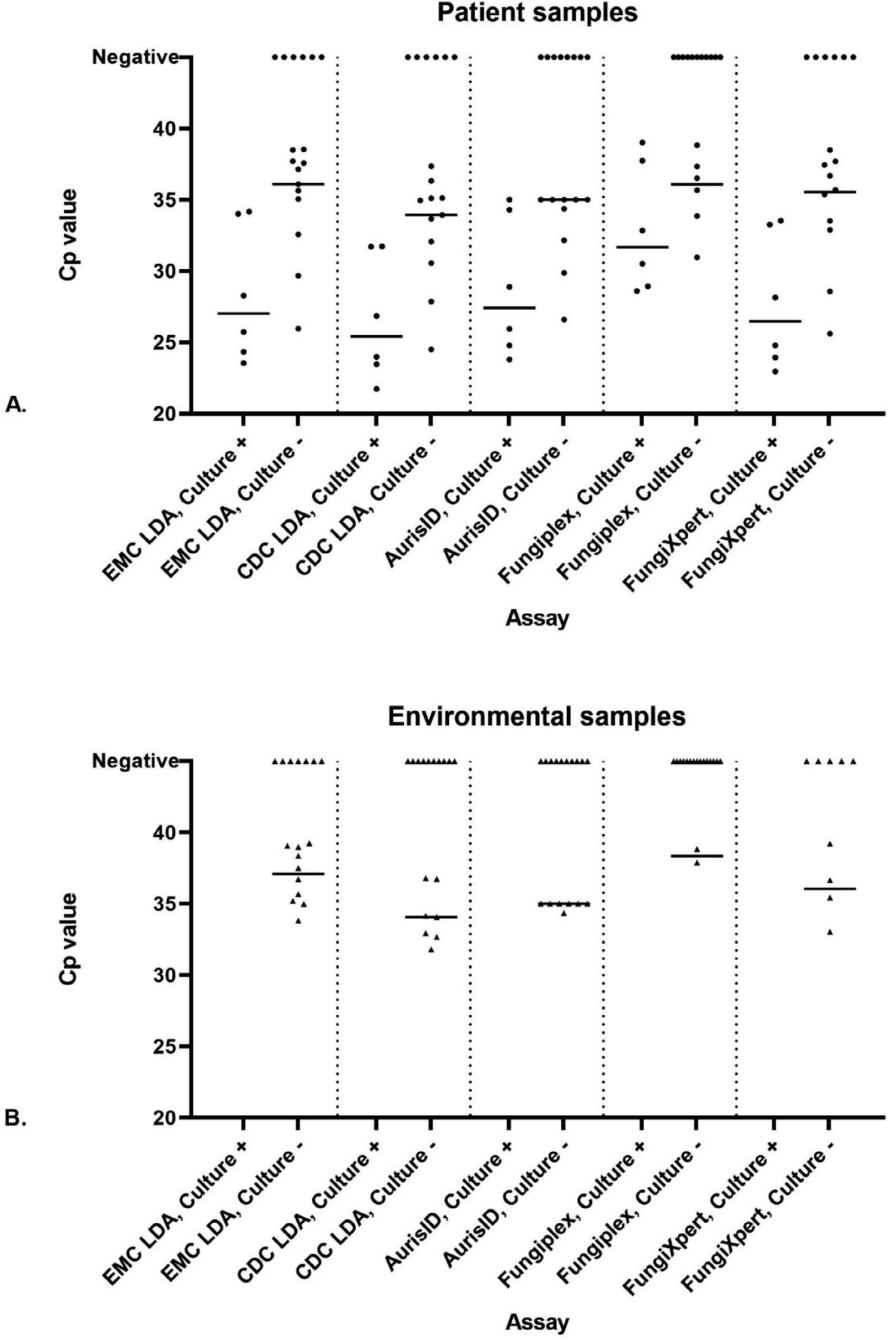
Comparative Cp‐values of five real‐time PCR assays for *C. auris* in culture‐positive and culture‐negative patients (A) and environmental (B) samples. −, *C. auris* culture negative samples; +, *C. auris* culture positive samples; LDA, Laboratory‐developed assay. Dots indicate individual Cp‐values, the horizontal line indicates the mean Cp‐value of a positive PCR. A Cp of 35 or above has higher uncertainty for the AurisID assay.

The positivity rates of culture and PCR assays on patient samples are displayed in Figure 2. Culture was positive in 6/23 patient samples. No environmental samples showed any growth. Out of the 23 patient samples, six were positive via both culture and PCR, while an additional sample was positive in at least one of the PCR tests. For the 17 environmental samples, none showed any growth on culture, but 10 were positive in at least one of the PCR tests. From the 40 negative environmental control swabs, none of the PCR assays tested positive. Table S2 details the 13 samples with culture negative, PCR positive results, including Cp‐values for each PCR assay. Finally, the Cp‐values of the various real‐time PCR assays were compared to culture. In general, the culture positive samples had lower Cp‐values than culture negative samples (Figure 3).

Compared to “any positive test”, the EMC LDA had the highest positivity rate, being positive in all instances where any test was positive. The CDC LDA had the second highest positivity rate, being positive in 17/17 patient samples and 7 out of 10 environmental samples. The AurisID was positive in 15/17 patient samples and 7/10 environmental samples with any positive test. The FungiXpert was positive in 15/17 patient samples and in 4/6 positive environmental samples; however, four environmental samples could not be tested with FungiXpert due to insufficient sample volume. The Fungiplex was positive in 12/17 patient samples and 2/10 environmental samples with any positive test.

Additionally, Figure 3 shows the Cp‐values of culture‐negative and culture‐positive patient and environmental samples for those with any positive test, with higher median Cq values for environmental samples.

## 4 Discussion

The ongoing spread of *C. auris* as a healthcare‐associated pathogen necessitates accurate and rapid detection methods. In this study, we compared the performance of three commercial real‐time PCR assays and two laboratory‐developed real‐time PCR assays for the detection of *C. auris* DNA in screening swabs. The lowest LOD with 8 conidia/reaction was found for the EMC LDA, while the CDC LDA, AurisID and FungiXpert were slightly less sensitive, with a LOD of 16–19 conidia/reaction. The results from the LOD experiments were in line with the results from the clinical sensitivity analysis, showing the highest positivity rate for the EMC LDA and the lowest positivity rate for the Fungiplex assay.

The LODs in our study differed from those reported in previous research, likely due to variations in standardisation, methodology, and reporting practices. In the present study, the CDC LDA demonstrated a LOD of 795 conidia/mL. Conversely, a previous study that assessed the LOD of the CDC LDA reported a LOD of 1 CFU/reaction (200 CFU/mL) (Table 1) [15]. The discrepancy between the CFU‐based and conidia‐count‐based LODs may be due to differences in quantification methods. We prepared the inoculum and quantified the conidia concentration using a Bürker‐Türk Counting Chamber. In contrast, previous studies have based their calculations on viable culturable colonies (colony forming units, CFU) or total extracted DNA (genomic copies). These methodological differences impede direct comparisons between our results and those of earlier studies, as well as among the previous studies themselves. CFU‐based methods are variable due to differences in viable culturable colonies when different culture methods are used. With genomic copies or counted conidia, the input in the PCR reaction is similar and thus more comparable. Additionally, variations in nucleic acid extraction procedures could contribute to the observed differences. However, by testing different assays using the same DNA sample, our study enables a direct comparison of their LODs and diagnostic sensitivity. Therefore, there is an urgent need for an international reference standard to accurately assess assay sensitivity, which is crucial for effective *C. auris* detection and global health.

The LOD of the AurisID assay was previously reported as 5 genomic copies per reaction, determined by measuring DNA concentration and calculating the number of genomic copies [18]. Here we report a LOD for this assay of 19 conidia per reaction, equivalent to 960 conidia/mL. Additionally, the same study reported a LOD of 50 genomic copies per reaction for the Fungiplex assay [18]. Our findings also indicated a higher LOD for the Fungiplex assay compared to the AurisID, with a LOD of 596 conidia per reaction.

To test the cross reactivity of the *C. auris* assays, we tested them against common *Candida* species, as well as more rare but closely related *Candida* species and some common fungal species that may be present in swabs from groin, axillary or nares. The PCR assays target fungal‐specific regions; therefore, cross‐reactivity is most likely to occur among closely related fungal species, which is why we focused our cross‐reactivity analysis exclusively on fungi. Most assays demonstrated high specificity for *C. auris* . However, both the CDC LDA and AurisID assays showed cross‐reactivity with *C. pseudohaemolonii*, consistent with previous findings [18]. Notably, while the AurisID assay previously cross‐reacted with *C. haemulonii* and *C. duobushaemulonii*, this was not observed in our study, possibly due to a lower DNA concentration used. Cross‐reactivity occurred only at high concentrations (5 × 10^5^ copies/reaction for *C. haemulonii* and *C. pseudohaemolonii*; 5 × 10^4^ copies/reaction for *C. duobushaemulonii*). Although colonisation by these yeast species is rare in most regions, it is imperative to confirm species identification through culture and subsequent methods such as MALDI‐TOF MS or sequence analysis. This is essential for distinguishing between the *C. haemulonii* species complex and *C. auris* sensu stricto, especially when using the CDC LDA or the AurisID. Accurate identification is particularly important in non/low‐endemic settings where unexpected PCR results may occur.

Interestingly, our study found that only 6 out of 17 (35%) *C. auris* PCR‐positive patient samples were culture positive. This contrasts sharply with findings from New York, where 545 out of 618 (88%) *C. auris* PCR‐positive samples were culture positive [19]. Our results are more similar to a study using the EMC LDA, which reported a culture positivity rate of 1 out of 7 (14%) compared to PCR positivity [17]. The New York study employed the CDC LDA, which is slightly less sensitive than the EMC LDA in our hands. Additionally, the New York study primarily tested swabs from nares and axillary/groin areas, which are typically colonised with higher CFU, thereby increasing the likelihood of culturing *C. auris* . In contrast, our study included a wider range of sample types. Furthermore, the culture methods used in the New York study, including both selective and non‐selective agar as well as enrichment broth, may have provided greater sensitivity compared to the methods used in our study. The optimal culture technique for *C. auris* remains to be determined and should be explored in a larger study in an endemic setting.

Several limitations of this study must be acknowledged. First, it was conducted in a non‐endemic region with a limited number of *C. auris* cases nationwide, resulting in a small sample size of *C. auris* ‐positive samples. Second, the study lacked a definitive gold standard; we used any assay‐positive result as a surrogate, which may have included false positives and affected benchmark accuracy. Although most samples tested positive with multiple assays, two samples were positive with only the EMC LDA, which had the lowest LOD and high Cp‐values, suggesting they were likely true positives. Confirmation through additional studies is needed to further validate and reinforce our findings.

## Author Contributions

**Jochem B. Buil:** conceptualization, methodology, writing – original draft, formal analysis. **Bart van den Bosch:** methodology, investigation, writing – review and editing, data curation, formal analysis. **Suzan J. van der Maas:** methodology, investigation, writing – review and editing, data curation. **Eelco F. J. Meijer:** conceptualization, writing – review and editing. **Theun de Groot:** conceptualization, writing – review and editing. **Joseph Meletiadis:** resources, writing – review and editing. **Paul E. Verweij:** conceptualization, writing – review and editing. **Willem J. G. Melchers:** conceptualization, methodology, writing – review and editing. **Suzan D. Pas:** conceptualization, methodology, supervision, writing – review and editing.

## Conflicts of Interest

J.B.B. reports grants from Gilead Sciences, BioMérieux, lectures for Gilead Sciences and Pfizer, and advisory board for Gilead Sciences; all grants are paid to the institute. E.F.J.M. discloses speaker engagements for Gilead Sciences, advisory board fees from Pfizer, and research funding from MundiPharma and Scynexis. S.J.M. reports grants from Gilead Sciences and Mundipharma. P.E.V. reports consulting fees, advisory board and speaker fees from Gilead Sciences and consulting fees from Mundipharma, all paid to the institute. All authors declare no conflicts of interest.

## Supporting information


**Table S1.** Results of the analytical specificity and sensitivity analysis.
**Table S2.** Overview of patient and environmental samples with negative culture and positive PCR results.

## Data Availability

The data that support the findings of this study are available from the corresponding author upon reasonable request.
