# Supplementary material for: Performance Evaluation of Five Real‐Time PCR Assays for the Detection of Candida auris DNA
Source: Mycoses. 2025 May 3;68(5):e70065. doi: 10.1111/myc.70065 (PMC12048891; doi:10.1111/myc.70065)
Supplement: Supplementary file 1 — Table S1. Results of the analytical specificity and sensitivity analysis. Table S2. Overview of patient and environmental samples with negative culture and positive PCR results. [file MYC-68-e70065-s001.docx]

**Table S1** Results of the analytical specificity and sensitivity analysis

| **Strain** | **Species** | **LDA (EMC)** | **LDA (CDC)** | **AurisID (OLM diagnostics)** | **FungiXpert® (Genobio)** | **Fungiplex® (Bruker)** |
| --- | --- | --- | --- | --- | --- | --- |
| **M.072 – 38** | *Candida auris* Clade I | POS  Cp 16.32 | POS  Cp 14.70 | POS  Cp 16.91 | POS  Cp 16.32 | POS  Cp 20.29 |
| **M.072 – 39** | *Candida auris* Clade I | POS  Cp 16.59 | POS  Cp 15.07 | POS  Cp 17.17 | POS  Cp 15.72 | POS  Cp 20.68 |
| **M.072 – 25** | *Candida auris* Clade II | POS  Cp 17.10 | POS  Cp 15.25 | POS  Cp 17.53 | POS  Cp 16.36 | POS  Cp 21.16 |
| **M.072 – 26** | *Candida auris* Clade II | POS  Cp 19.83 | POS  Cp 14.18 | POS  Cp 16.57 | POS  Cp 16.84 | POS  Cp 21.18 |
| **M.072 – 29** | *Candida auris* Clade III | POS  Cp 17.82 | POS  Cp 16.36 | POS  Cp 18.21 | POS  Cp 17.82 | POS  Cp 21.60 |
| **M.072 – 30** | *Candida auris* Clade III | POS  Cp 15.86 | POS  Cp 14.87 | POS  Cp 17.07 | POS  Cp 17.10 | POS  Cp 20.82 |
| **M.072 – 28** | *Candida auris* Clade IV | POS  Cp 17.66 | POS  Cp 16.01 | POS  Cp 18.55 | POS  Cp 17.30 | POS  Cp 21.61 |
| **M.072 – 31** | *Candida auris* Clade IV | POS  Cp 16.64 | POS  Cp 15.09 | POS  Cp 17.55 | POS  Cp 16.78 | POS  Cp 20.61 |
| **M.072 – 36** | *Candida auris* Clade V | POS  Cp 16.67 | POS  Cp 15.26 | POS  Cp 17.13 | POS  Cp 16.31 | POS  Cp 21.23 |
| **M.072 – 37** | *Candida auris* Clade V | POS  Cp 17.11 | POS  Cp 14.78 | POS  Cp 16.99 | POS  Cp 16.71 | POS  Cp 21.35 |
| **M.034 – 27** | *Candida haemulonii* | NEG | NEG | NEG | NEG | NEG |
| **v309-55** | *Candida duobushaemulonii* | NEG | NEG | NEG | NEG | NEG |
| **v294-54** | *Candida pseudohaemulonii* | NEG | POS  Cp 37.76 | POS  Cp 35.00 | NEG | NEG |
| **v258-07** | *Candida blankii* | NEG | NEG | NEG | NEG | NEG |
| **M.029 – 21** | *Clavispora lusitanieae* | NEG | NEG | NEG | NEG | NEG |
| **M.076 – 8** | *Candida albicans* | NEG | NEG | NEG | NEG | NEG |
| **M.075 – 10** | *Candida parapsilosis* | NEG | NEG | NEG | NEG | NEG |
| **M.053 – 41** | *Pichia kudriavzevii* | NEG | NEG | NEG | NEG | NEG |
| **M.038 – 70** | *Kluyveromyces marxianus* | NEG | NEG | NEG | NEG | NEG |
| **M.050 – 43** | *Geotrichum candidum* | NEG | NEG | NEG | NEG | NEG |
| **M.009-54** | *Trichophyton rubrum* | NEG | NEG | NEG | NEG | NEG |
| **v155-65** | *Trichophyton interdigitale* | NEG | NEG | NEG | NEG | NEG |

POS: positive, NEG: negative

**Table S2 Overview of patient and environmental samples with negative culture and positive PCR results**

| **Sample** |  | **Culture** | **LDA (EMC)** | **LDA (CDC)** | **AurisID (OLM diagnostics)*** | **FungiXpert® (Genobio)** | **Fungiplex® (Bruker)** |
| --- | --- | --- | --- | --- | --- | --- | --- |
| Patient: Axilla/groin swab | *C. auris* colonization status unknown. Patient in ICU during *C. auris* outbreak | NEG | POS  Cp 38.53 | POS  Cp 34.95 | NEG | POS  Cp 37.45 | NEG |
| Patient: Wound foot swab | Patient with proven *C. auris*  colonization^ | NEG | POS  Cp 37.57 | POS  Cp 35.11 | POS  Cp 35.00 | POS  Cp 36.67 | NEG |
| Patient: Axilla/groin swab | *C. auris* colonization status unknown. Patient in ICU during *C. auris* outbreak | NEG | POS  Cp 38.49 | POS  Cp 35.12 | POS  Cp 35.00 | POS  Cp 35.37 | NEG |
| Patient: Ear swab | Patient with proven *C. auris*  colonization^ | NEG | POS  Cp 37.14 | POS  Cp 36.32 | NEG | POS  Cp 38.49 | NEG |
| Patient: Axilla/groin swab | Patient with proven *C. auris*  colonization^ | NEG | POS  Cp 37.70 | POS  Cp 37.35 | POS  Cp 35.00 | NEG | NEG |
| Environmental swab: Stethoscope | Room of a patient with proven *C. auris* colonization^ | NEG | POS  Cp 35.20 | POS  Cp 32.67 | POS  Cp 35.00 | POS  Cp 35.44 | NEG |
| Environmental swab: Dispenser | Room of a patient with proven *C. auris* colonization^ | NEG | POS  Cp 35.68 | POS  Cp 34.14 | POS  Cp 35.00 | POS  Cp 36.66 | NEG |
| Environmental swab: ear thermometer | Room of a patient with proven *C. auris* colonization* | NEG | POS  Cp 36.72 | POS  Cp 34.06 | POS  Cp 35.00 | Not enough sample | NEG |
| Environmental swab: floor | Room of a patient with proven *C. auris* colonization^ | NEG | POS  Cp 37.50 | POS  Cp 36.81 | NEG | Not enough sample | NEG |
| Environmental swab: light switch patient room | Room of a patient with proven *C. auris* colonization^ | NEG | POS  Cp 38.35 | NEG | POS  Cp 35.00 | POS  Cp 39.22 | NEG |
| Environmental swab: Intercom | Room of a patient with proven *C. auris* colonization* | NEG | POS  Cp 38.99 | NEG | NEG | NEG | NEG |
| Environmental swab: touchscreens pumps | Room of a patient with proven *C. auris* colonization ^ | NEG | POS  Cp 39.07 | POS  Cp 36.76 | POS  Cp 35.00 | NEG | NEG |
| Environmental swab: chair | Room of a patient with proven *C. auris* colonization^ | NEG | POS  Cp 39.26 | NEG | NEG | Not enough sample | NEG |

Cp: Crossing point value *Cp of 35 or above has higher uncertainty for AurisID assay. ^Patient was culture proven on swabs from a wound.
